# Elevated Serum Follistatin-Like Protein 1 Levels Correlate with Disease Activity and Severity in Behçet’s Disease: A Case–Control Study

**DOI:** 10.31138/mjr.240825.ral

**Published:** 2026-06-01

**Authors:** Sara Farrag, Nesreen I. Ibrahim Ismail, Maha G. Abd El-Kader, Eman M. Shawky

**Affiliations:** 1 Department of Rheumatology, Rehabilitation and Physical Medicine, Faculty of Medicine, Assiut University, Assiut, Egypt;; 2 Department of Clinical Pathology, Faculty of Medicine, Assiut University, Assiut, Egypt

**Keywords:** Behçet syndrome, follistatin-like protein 1, biomarkers, disease severity, inflammation, enzyme-linked immunosorbent assay

## Abstract

**Objective:**

Behçet’s disease (BD) is chronic multisystem inflammatory condition with wide spectrum of clinical manifestations, lacking specific laboratory biomarkers for diagnosis or monitoring. Follistatin-like protein 1 (FSTL1), having dual roles in inflammation, remains largely unexplored in BD. The present study aimed to evaluate FSTL1 levels in the serum of BD patients and investigate their association with disease activity and severity.

**Methods:**

This prospective, hospital-based case-control study included 40 BD patients, the control group consisted of 40 healthy individuals age and sex matched with the patients. FSTL1 concentrations were measured using a double-antibody sandwich ELISA. The disease activity and cumulative disease related damage were assessed using the Behçet’s Disease Current Activity Form (BDCAF), and the Behçet’s Syndrome Overall Damage Index (BODI).

**Results:**

The median serum FSTL1 concentrations were significantly elevated in BD patients in comparison to controls :4.05 (1.9 – 9.1), 3.55 (2.2 – 4.8) ng/ml; p = 0.024, r (rank-biserial correlation) = 0.036 represent very small effect size. FSTL1 levels also showed moderate positive correlations with both BDCAF (r = 0.397) and BODI (r = 0.391) scores, suggesting that higher FSTL1 levels reflect increased inflammatory activity and tissue involvement.

**Conclusion:**

Our study demonstrates an elevation in serum FSTL1 levels in BD and positively correlate with disease BD activity and cumulative damage. FSTL1 may reflect inflammatory burden in BD, but further validation is needed to establish causality.

## INTRODUCTION

Behçet’s disease (BD) is an autoinflammatory chronic disorder with recurrent vasculitic involvement affecting multiple organs.^1^ Clinical manifestations of BD include recurrent oral aphthae, uveitis, skin eruptions, vascular, nervous system, and bowel manifestations.^2,3^ The exact aetiology of BD is still unclear, although genetic predispositions and environmental triggers are believed to contribute to its development.^4^

Most of Behçet’s disease manifestations have features of systemic perivasculitis. Cell-mediated immunity, especially Th1-driven responses, plays pivotal role in BD pathogenesis, Activated Th1 cells amplify T-lymphocyte counts and release proinflammatory cytokines that drive clinical manifestations and may reflect disease activity. Enhanced macrophage activation, intense neutrophil chemotaxis, and phagocytic activities are also noted in BD lesions. These proinflammatory cytokines may be used as indicators of disease severity.^4,5^ Indeed, studying biomarkers that may drive inflammatory pathways involved in BD, is consider a research priority.

Follistatin-like proteins (FLP) are acidic cysteine-rich secreted glycoproteins of the SPARC family share structural homology with follistatin.^6^ Among five iso-forms (FSTL1–5), FSTL1 is produced by mesenchymal cells and participates in several signalling and regulatory processes. Experimental studies have shown that FSTL1 has inflammatory and anti-inflammatory effects, depending on the biological context.^7,8^ During the acute phase, these proteins may function in protective manner as anti-inflammatory agents; whereas in chronic states, it promotes inflammation.^9^ FSTL1 is upregulated by multiple innate immune system stimuli and proinflammatory cytokines including TNF alpha, IL-6 and IL-1b.^10^ FSTL1 increases the responsiveness of macrophages, monocytes and T cells to inflammatory stimuli, with subsequent increase in the expression of multiple cytokines (IL-1b, TNF alpha, IL-6), in addition to several chemokines (CCL2/MCP and CXCL8/IL-8).^11,12^ Moreover, increased expression of FSTL1 triggered activation of the NLRP3 pathway in macrophages and monocytes and promoted release of IL-1β and related cytokines involved in inflammation.^13^

Regarding its anti-inflammatory role, FSTL1 has been reported to positively affect endothelial cell responses, leukocyte adhesion, and tissue repair, suggesting that under certain conditions it may preserve vascular integrity.^14,15^ In the setting of systemic vasculitis such as Behçet’s disease, this dual behaviour could influence both vascular inflammation and healing dynamics. Further research is warranted to specify whether the observed increase in circulating FSTL1 in BD represents a compensatory anti-inflammatory response or a marker of ongoing vascular injury.

Elevated circulating FSTL1 concentrations were found in rheumatoid arthritis (RA), osteoarthritis, Sjogren’s syndrome, and had positive correlation with RA, juvenile idiopathic arthritis (JIA), and systemic lupus erythematosus (SLE) disease activity indices.^10,16,17^ Thus, FSTL1 could be an important mediator linking both immune and vascular inflammation. It is upregulated by IL-1β, TNF alpha, and TGF beta cytokines known to be involved in BD pathogenesis, with subsequent amplification of IL-1β signalling in monocytes and macrophages. FSTL1, also expressed in vascular smooth muscle cells, endothelium with subsequent affection of endothelial dysfunction and vascular remodelling,^14,15^ which are considered key pathological features of BD, suggesting that FSTL1 may not merely represent an inflammatory marker but may also reflect processes underlying immune activation and vascular injury in BD. This provided the rationale for evaluating FSTL1 serum levels and their relationship to disease activity and severity in the current study.

Therefore, this study aimed to evaluate serum FSTL1 levels in BD patients and to investigate its relationships to BD clinical characteristics, laboratory parameters, disease activity, and severity.

## PATIENTS AND METHODS

### Study Design and Participants

This prospective, hospital-based case-control study enrolled 40 adults meeting the (ICBD): International Team for the Revision of the International Criteria for Behçet’s Disease.^18^ Data were collected between May 2024 and December 2024 at the Rheumatology and Rehabilitation Clinics, University Hospitals. Forty age- and sex-matched healthy individuals enrolled as controls. Controls were selected from hospital staff and volunteers without autoimmune or inflammatory disorders. Exclusion criteria included individuals with other autoimmune diseases, infections, malignancies, and patients who refused to participate, to minimise confounding and selection bias. Primary outcome variable: serum FSTL1 level (ng/ml). Main explanatory variables: disease activity (BDCAF score) and cumulative damage (BODI score).

### Ethical Considerations

The study was approved by the Institutional Review Board and Ethical Committee of the Faculty of Medicine on April, 2024, Approval No (IRB No.: 04-2024-300501) in compliance with the Helsinki Declaration. All participants provided written informed consent prior to inclusion, and after receiving an explanation regarding the study’s purpose, procedures and their right to withdraw from the study at any time without any negative consequences.

### Sample Size Estimation

Using previously reported mean ± SD of FSTL1 values in BD versus controls,^19^ OpenEpi v3 calculated that 80 total participants (40 per group) would provide 95% power at α = 0.05 for a two-tailed t-test.

### Clinical Evaluation

All subjects underwent a detailed history, including demographics, disease duration, symptom history, medications, and family history, and a comprehensive physical examination with emphasis on mucocutaneous, ocular, neurological, and vascular signs. An ophthalmologist evaluated every participant. Main explanatory variables: (BDCAF score) and (BODI score).

*Activity Assessment:* (BDCAF) scores symptoms over the past 4 weeks (e.g., ulcers, skin lesions, arthralgia, gastrointestinal (GI), central nervous system (CNS), vascular and ocular), scoring only new or worsening features; total range 0–12, with active disease defined as a score ≥2.^20^

*Damage Assessment:* (BODI) records permanent organ damage persisting ≥ 6 months, regardless of whether it was directly attributed to BD; each item scores 1 point, with subitems weighted for severity.^21^

### Serum FSTL1 level measurement

Primary outcome variable: serum FSTL1 level (ng/ml). Blood (4 ml) was collected into separator tubes for the serum, was allowed to be clotted for about 30 mins at room temperature, and then centrifuged at 3,000 rpm for 20 min. Serum was aliquoted, stored at –20 °C. FSTL1 concentrations were measured using double-antibody sandwich ELISA (SunRed Biotech, Shanghai; catalog 201-12-2703) per manufacturer’s instructions; results are presented in ng/ml.

### Statistical Analysis

SPSS version 26 was used. Normally distributed continuous variables presented as mean ± SD; non-normally distributed data as median (minimum-maximum), and categorical data as frequencies (%). Independent-samples t-test or Mann–Whitney U, and χ^2^(chi-square) or Fisher’s exact test for categorical variables were used for comparison. Spearman’s correlation was used to assess associations. A two-tailed p < 0.05 was considered significant.

This study was conducted according to the STROBE (Strengthening the Reporting of Observational Studies in Epidemiology) guidelines, following EQUATOR Network recommendations.

## RESULTS

### Demographics and Clinical Features

In BD patients, disease duration the median value was 6 (2 – 20) years. Mucocutaneous ulcers were present in most cases: oral (65%) and genital (95%), with ocular involvement in 67.5%. Musculoskeletal, neurological, and vascular manifestations occurred in 15%, 52.5%, and 55%, respectively. Mean BDCAF and BODI scores were 3.4 ± 1.82 and 6.88 ± 2.15 (**Table 1**).

**Table 1.** Demographic and clinical data of Behçet’s disease patients and control group.

**Demographic Data**	**Behçet’s disease Group (n = 40)**	**Control Group (n = 40)**	**P-value**

Age (years)	35 (25 – 60)	33 (18 – 58)	0.300

Gender			0.770
• Female	8 (20%)	6 (15%)	
• Male	32 (80%)	34 (85%)	

Disease duration (yrs)	6 (2 – 20)	_	

Smoker status			
• Non-smoker			
• Smoker	23 (57.5%)	21 (52.5%)	0.748
• Ex-smoker	14 (35%)	17 (42.5%)	
	3 (7.5%)	2 (5%)	

BMI	24.05 (19.7 – 35.1)	25.05 (21.5 – 34)	0.087

**Clinical Characteristics**			

Oral ulcers	26 (65%)	_	_

Genital ulcers	38 (95%)	_	_

Ocular manifestations	27 (67.5%)	_	_

Musculoskeletal manifestations	6 (15%)	_	_

Neurological manifestations	21 (52.5%)	_	_

Vascular manifestations	22 (55%)	_	_

BDCAF	3.4 ± 1.82	_	_

BODI	6.88 ± 2.15	_	_

Data expressed as mean ±SD or median (Min-Max) and N (%). Significance defined by p < 0.05 BD: Behçet’s Disease; BDCAF: Behcet’s Disease Current Activity Form; BODI: Behçet’s syndrome overall damage index.

### Therapeutic Regimens

As shown in **Table 2**, the most frequently administered drugs among Behçet’s disease patients were azathioprine and corticosteroids, followed by methotrexate and cyclophosphamide.

**Table 2.** Current treatment of Behçet’s disease patients.

**Drug**	**Behçet’s disease Group (n = 40)**
Azathioprine	32 (80%)
Corticosteroids	32 (80%)
Methotrexate	29 (72.5%)
Cyclophosphamide	16 (40%)
Adalimumab	11 (27.5%)
Anticoagulant therapy	7 (17.5%)
Cyclosporine	1 (2.5%)

Data expressed as N (%).

### Laboratory Findings

Median (Min-Max) hematologic and biochemical parameters for the studied groups are presented in **Table 3**.

**Table 3.** Haematologic and biochemical parameters for Behçet’s disease patients and control group.

**Variable**	**Behçet’s disease Group (n = 40)**	**Control Group (n = 40)**	**P-value**
**Haemoglobin (g/dl)**	13.35 (11 – 17)	12.5 (10 – 17)	0.101
**Leucocytes (10^3^/μl )**	6.29 (3.26 – 13.25)	5.9 (3.8 – 17.5)	0.962
**Platelets (10^3^/μl )**	284 (150 – 626)	253.5 (179 – 500)	0.179
**Serum Urea (mmol/L)**	4.1 (2.6 – 35)	4.05 (1 – 26)	0.718
**Serum Creatinine (μmol/L)**	65.15 (33 – 110)	71 (20 – 97.2)	0.109
**ESR (mm/h)**	40 (2 – 100)	6 (5 – 22)	<0.001*
**AST (U/L)**	22 (12 – 93)	21 (10 – 76)	0.885
**ALT (U/L)**	18 (5 – 79)	20 (5 – 62)	0.586

Data expressed as median (Min-Max). Significance is defined by P < 0.05. BD: Behçet’s Disease; ESR: erythrocyte sedimentation rate; AST: aspartate transaminase; ALT: alanine transaminase; FSTL1: Follistatin like protein 1.

### FSTL1 Group Comparison

**Figure 1.**
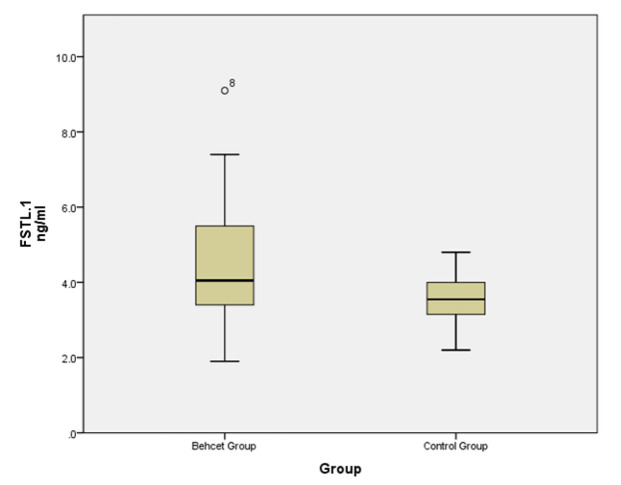
**(above).** Boxplot diagram for FSTL1 values among studied patients. BD patients had significantly higher FSTL1 levels [median 4.05 (1.9 – 9.1), 3.55 (2.2 – 4.8) ng/ml; than controls 3.55 (2.2 – 4.8) ng/ml; p = 0.024, Mann–Whitney U test), indicating increased systemic inflammation in BD.

In addition to the significant difference in median serum FSTL1 levels between BD patients [4.05 (1.9 – 9.1), the 95% confidence interval CI (3.5 – 4.5)] and controls: [3.55 (2.2 – 4.8) the 95% CI 3.3 – 3.95ng/ml; p = 0.024], illustrated in **Table 4**, the r (rank-biserial correlation) = 0.036, and the 95% CI for the difference indicating a moderate effect size, suggesting a clinically meaningful elevation of FSTL1 among BD patients. This reinforces that the difference is not only statistically significant but also relevant in magnitude (**Figure 1**).

**Table 4.** Comparison of studied clinical variables with FSTL1 level in Behçet’s disease patients.

**Organ Involvement**	**n**	**Serum FSTL1 biomarker**	

		**Median (Min-Max)**	**P value**

Oral Ulcers			

· No	14	3.45 (1.9 – 9.1)	
95% CI (Median)		3.0 – 4.0	0.023*

· Yes	26	4.45 (1.9 – 7.4)	
95% CI (Median)		3.9 – 5.99	

Genital Ulcers			

· No	2	5.45 (4.4 – 6.5)	
95% CI (Median)			0.282

· Yes	38	4.00 (1.9 – 9.1)	
95% CI (Median)		3.45 – 4.5	

Ocular manifestations			

· No	13	3.7 (2.4 – 9.1)	
95% CI (Median)		3.0 – 4.8	0.441

· Yes	27	4.2 (1.9 – 7.4)	
95% CI (Median)		3.5 – 5.0	

Musculoskeletal manifestations			

· No	34	4.05 (1.9 – 7.4)	
95% CI (Median)		3.4 – 4.8	0.541

· Yes	6	4.1 (3.5 – 9.1)	
95% CI (Median)			

Neurological manifestations			

· No	19	4.3 (1.9 – 7.4)	
95% CI (Median)		3.7 – 5.8	0.138

· Yes	21	3.5 (1.9 – 9.1)	
95% CI (Median)		3.1 – 4.4	

Vascular manifestations			

· No	18	4.35 (2.9 – 9.1)	
95% CI (Median)		3.35 – 6.0	0.411

· Yes	22	3.85 (1.9 – 7.4)	
95% CI (Median)		3.4 – 4.8	

Data expressed as median (Min-Max) and 95% CI (confidence intervals). Significance is defined by P < 0.05. FSTL1: Follistatin like protein 1.

### Subgroup Analyses

Gender differences: Males tended to have slightly higher FSTL1 levels than females (5.83 vs 4.88 ng/ml in males; 4.03 vs 4.10 ng/ml in females). However, the small sample size limits interpretation.

Steroid use:  FSTL1 median levels did not differ significantly between patients receiving corticosteroid and those who were not [4.25(1.9–7.4)] than non-users [3.3(2.5–9.1)]ng/ml.

Biologic therapy: No difference was found between biologic and conventional therapy groups regarding FSTL1 serum level.

**Figure 2.**
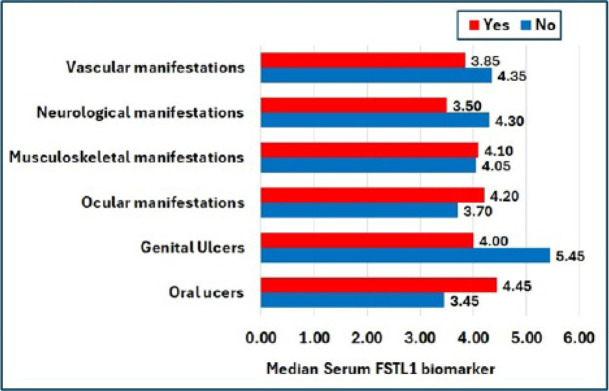
**(below)**. Comparison of serum FSTL1 levels among Behçet’s disease patients according to clinical manifestations. Bars representing median FSTL1 values (ng/mL). FSTL1 levels were significantly higher in patients with oral ulcers compared to those without (p = 0.023), while no significant differences were observed for other clinical features. Statistical comparisons were performed using the Mann–Whitney U test.

We analysed the difference between the median values for FSTL1 levels and different BD clinical manifestations, and no statistically significant difference was found except for patients with oral ulcers (p =0.023). This finding may indicate a possible link between mucosal inflammation and FSTL1 expression (**Table 4, Figure 2**).

**Figure 3.**
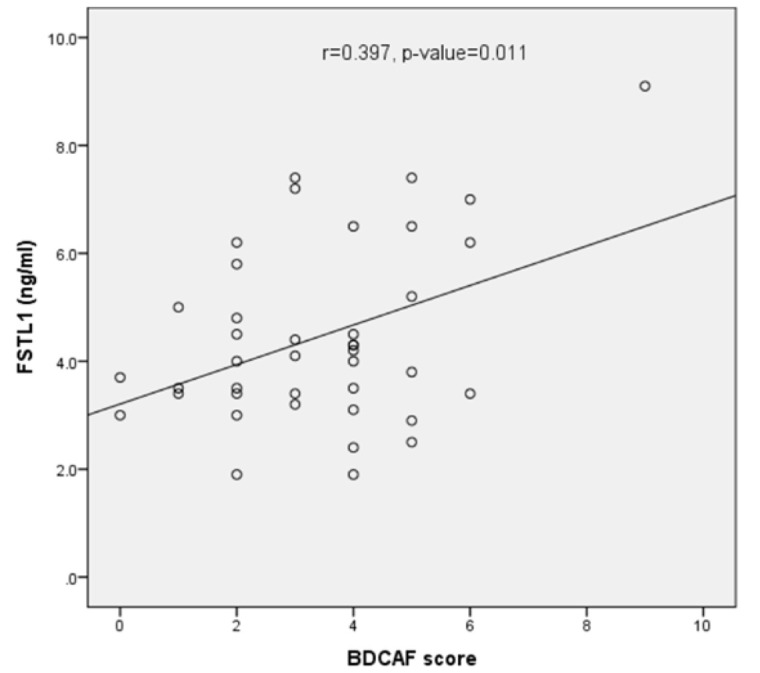
**(above).** Scatter diagram for correlation between FSTL1 and BDCAF, among studied patients. Statistically significant positive correlation between BDCAF score and serum level of FSTL1, as an increase in BDCAF score is associated with an increase in serum FSTL1 (r=0.397, p value=0.011).

**Figure 4.**
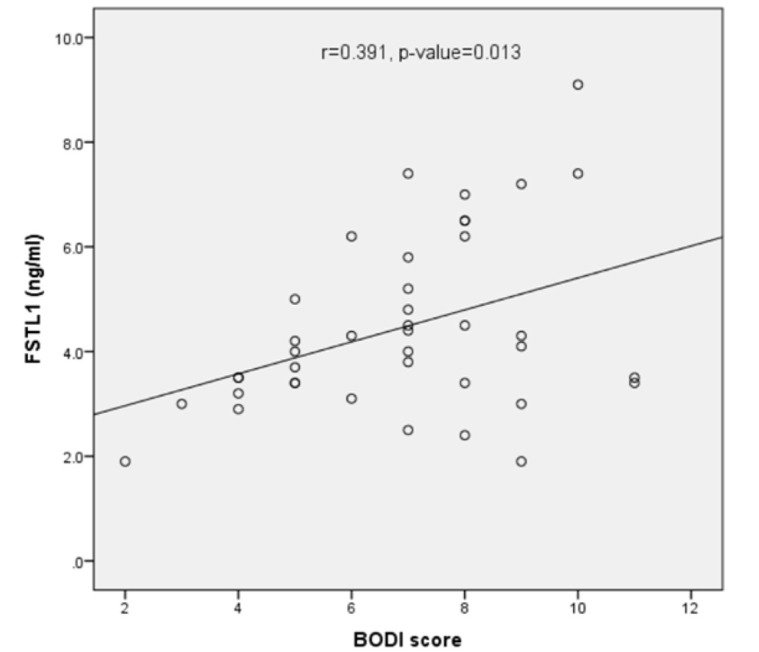
**(below).** Scatter diagram for correlation between FSTL1 and BODI, among studied patients. Statistically significant, positive correlation between BODI score and serum level of FSTL1, as an increase in BODI score is associated with an increase in serum FSTL1 (r=0.391, p value=0.013).

To analyse the relation between FSTL1 and BD activity and severity, correlation analysis was done**.** Serum FSTL1 levels showed moderate positive correlations with both (BDCAF; r = 0.397; p = 0.011) and (BODI; r = 0.391; p = 0.013) as shown in (**Figures 3 and 4**) and detailed in (**Table 5**). These findings indicate that higher FSTL1 levels reflect increased inflammatory activity and cumulative organ involvement, supporting its possible value as a biomarker for BD monitoring beyond statistical associations alone.

**Table 5.** Correlation of age and clinical/laboratory characteristics with FSTL1 level.

**Variable**	**r**	**P-value**
**Age**	0.123	0.276
**Disease duration**	0.067	0.683
**ESR**	0.033	0.839
**BDCAF**	0.397	**0.011**
**BODI**	0.391	**0.013**

Significance is defined by P < 0.05. FSTL1: Follistatin like protein 1; r: correlation coefficient, ESR: erythrocyte sedimentation rate; BDCAF: Behcet’s Disease Current Activity Form; BODI: Bechet’s syndrome overall damage index.

**Figure 5.**
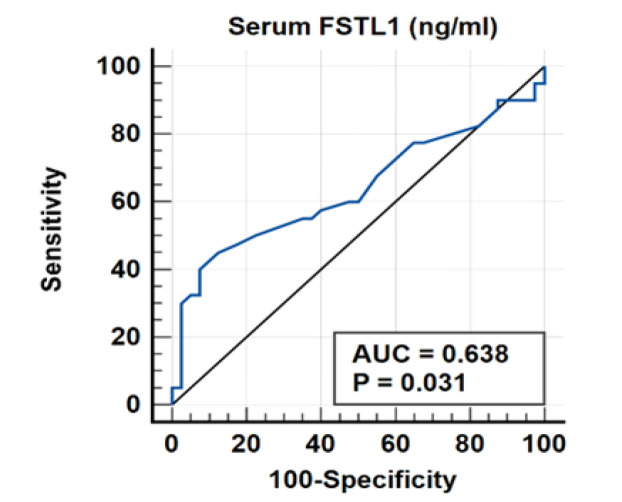
ROC curve of Serum FSTL1 for diagnosis of patients with Behçet disease. The best cut-off value (>4.3 ng/mL) yielded 40% sensitivity and 92.5% specificity, (AUC = 0.64, p = 0.03), suggesting potential use in multi-marker diagnostic panels rather than as a standalone biomarker.

**Table 6** presents stepwise multiple linear regression model to identify the main determinants of serum FSTL1 levels in BD patients, showing BDCAF and BODI as significant predictors, explaining 26.8% of the variance (adjusted R^2^ = 0.229, p = 0.003). Both predictors show positive associations, and the absence of multicollinearity supports their independent contributions. ROC curve analysis was used to assess the predictive ability of serum FSTL1 levels for the diagnosis of BD. At a cutoff value > 4.3 ng/ml, the area under the ROC curves (AUC) was 0.64 with a sensitivity of 40%, specificity of 92.5%. and accuracy 66.5% (95% Confidence interval 0.052–0.74; p-value 0.03) (**Figure 5**). However, patients with FSTL1 levels above this cut-off tended to have higher BDCAF and BODI scores, indicating that elevated FSTL1 may reflect more inflammation and cumulative damage rather than a specific diagnostic marker. Therefore, the potential value of FSTL1 may help in disease monitoring or prognosis, rather than BD diagnosis.

**Table 6.** Stepwise multiple linear regression analysis examining predictors of FSTL1 levels among BD patients.

**Predictor**	**SE**	**β (Standardised Coefficient)**	**t**	**p-value**	**95% CI (Lower, Upper)**
**Constant**	0.86	-	1.83	**0.07**	(−0.169, 3.298)
**BDCAF**	0.13	0.33	2.42	**0.05**	(0.051, 0.584)
**BODI**	0.11	0.34	2.36	**0.02**	(0.038, 0.489)

Model Fit Statistics**: R^2^ = 0.268, Adjusted R^2^ = 0.229, F (2,37) = 6.780, p = 0.003; BDCAF: Behcet’s Disease Current Activity Form; BODI: Bechet’s syndrome overall damage index.

## DISCUSSION

(BD) is a chronic multisystem inflammatory vasculopathy of uncertain aetiology, diagnosis is still depending on characteristic clinical findings due to the absence of specific laboratory markers.^22^ Lacking objective tools for disease activity, prognostic indicators, and treatment monitoring tools has driven the research for reliable biomarkers in BD. Therefore, the need to detect serum biomarkers that may improve disease diagnosis, disease activity assessment and prognosis is crucial. This study addresses this knowledge gap, by investigating (FSTL1), a secreted glycoprotein of the SPARC family with established roles in inflammation, as a potential biomarker in BD. We measured serum FSTL1 levels in BD, evaluated their association with clinical phenotypes, BD disease activity, and tissue damage indices. Our findings demonstrate significantly elevated FSTL1 levels in BD patients compared to healthy controls [4.05 (1.9 – 9.1), 3.55 (2.2 – 4.8) ng/ml; p = 0.024] respectively, with significant positive correlations to disease activity and severity scores.

The observed elevation of FSTL1 level in BD can be understood through its dual role in inflammation. FSTL1 functions as both a damage-associated molecular pattern (DAMP) molecule and an active mediator of inflammatory signalling pathways.^8,23^ During acute inflammation, it may exert an anti-inflammatory function; however, in chronic inflammation, as in BD, it exhibits pro-inflammatory effects via multiple key mechanisms.^24^

FSTL1 increases the responsiveness of immune cells to inflammatory stimuli through activating the NLRP3 inflammasome, with a subsequent increase in IL-1β production, which has a central role in to BD pathogenesis.^13^ Moreover, FSTL1 also promotes fibrosis and inflammation through modulation in TGF-β/BMP signalling pathways,^25^ enhancing the production of some inflammatory chemokines and cytokines (MCP-1, IL-6, TNF-α) which are dysregulated in BD.^26^

Although the difference in serum levels of FSTL1 among corticosteroid users and non-users was not statistically significant, higher FSTL1 in corticosteroid treated patients may reflect the association of corticosteroid use with more active or severe disease or a potential direct effect of corticosteroids on FSTL1 expression. There was no difference in FSTL1 levels between patients receiving conventional therapy and those on biological agents. Data on biologic therapy were limited and did not show a clear pattern in FSTL1 levels. Most patients were not on biologics at the time of the study.

Preclinical study has demonstrated that FSTL1 neutralising antibodies may reduce the severity of inflammatory arthritis and may decrease pro-inflammatory cytokine production in mouse models.^24^ FSTL1 has been reported to preserve vascular integrity, and tissue repair, under certain conditions it may^14,15^ (both involved in BD pathogenesis), suggesting that FSTL1 may also have some potential therapeutic outcomes in BD, warranting future investigation of FSTL1-targeted interventions.

In the current study, serum FSTL1 level was higher in BD patients with oral ulcers compared to those without (p = 0.023 oral aphthae are BD most predominant and characteristic manifestations. The observed elevated FSTL1 level in these patients may reflect localised innate immune system activation, as FSTL1 is upregulated by IL-1β and TNF-α with subsequent increase in inflammatory cytokines secretion by macrophages and fibroblasts, response.^16^ The previous findings may suggest that FSTL1 may affect mucosal inflammation, further longitudinal studies during periods of ulcer flares and remission are needed for confirmation.

The observed positive correlations between FSTL1 and both BDCAF (r = 0.397, p = 0.011) and BODI (r = 0.391, p = 0.013) scores indicate that FSTL1 reflects not only active inflammatory processes but also cumulative organ damage, a property that could be particularly valuable for monitoring disease course and therapeutic response.

The stepwise regression analysis to identify the main determinants of serum FSTL1 was performed and we find that both BDCAF and BODI were the strongest independent predictors of FSTL1 levels elevation in BD patients, accounting for approximately 27% of the variance (adjusted R^2^ = 0.229, p = 0.003), suggesting that FSTL1 may be used as a biomarker for the current inflammatory activity and cumulative organ damage, consistent with FSTL1 established role immune system dysregulation in BD. Variables such as age, disease duration, clinical manifestations (e.g., ocular or vascular involvement) and ESR did not contribute significantly, possibly due to small sample size or their mediation through overall severity and damage indices. These sample limitations support further investigation of FSTL1 serum levels in larger, longitudinal studies including treatment naïve BD patients to explore both causality and unmeasured confounders.

The ROC analysis revealed that FSTL1 has modest diagnostic accuracy (AUC = 0.64) for discriminating BD patients from controls, with 40% sensitivity and 92.5% specificity at a cutoff of >4.3 ng/ml. While these values indicate that FSTL1 alone is insufficient to serve as a standalone diagnostic biomarker, the high specificity may suggest its potential utility as part of a composite biomarker panel.

Therefore, the potential value of FSTL1 may help in disease monitoring or prognosis, rather than BD diagnosis.

Our findings align with previous research examining FSTL1 in autoimmune diseases. Li et al. demonstrated elevated FSTL1 levels across multiple systemic autoimmune diseases including RA, systemic sclerosis, and SLE, establishing FSTL1 as a common inflammatory mediator in autoimmune conditions.^16^

Three cohorts of liver diseases patients by Li W et al.^27^ showed a significantly elevated FSTL 1 levels, with an AUC of 0.85 (95% CI, 0.75–0.96).

Similarly, Wilson et al. showed significant elevation in FSTL1 level with positive correlations between FSTL1 levels and disease activity in JIA^10^ and Wang et al. stated that FSTL1 may reflect the degree of joint affection in osteoarthritis, monitors disease progression, and assesses treatment effectiveness,^28^ which support our findings regarding the association between FSTL1 and disease activity tools.

Our results are comparable with previous BD study by Demir et al.,^19^ in demonstrating elevated serum FSTL1 in BD, the variations in absolute FSTL1 values between studies may reflect methodological factors such as ELISA kit differences, sample handling protocols (e.g., centrifugation speed, storage temperature), and timing of sample collection relative to disease flares. Also, population characteristics may contribute to that difference, including disease severity distribution (e.g., higher proportion of vascular/neurological involvement in our study), treatment regimens (80% corticosteroids in our study) and genetic/ethnic variations.

Our study has several novel contributions. First, we present serum FSTL1 levels from the sera of a well-defined, prospectively recruited BD cohort and quantify the magnitude of the difference (r=0.036) with ageand sex-matched controls. Second, we show moderate correlations of FSTL1 with validated disease activity and damage indices, strengthen the concept that FSTL1 level may reflects the current inflammatory status and cumulative tissue damage. Third, our subgroup analyses (treatment and organ-specific patterns) and together with adherence to STROBE guidelines support the methodological transparency. Finally, our study reports ROC performance metrics (AUC = 0.64; specificity 92.5% at >4.3 ng/ml), Altogether, these elements adding for future research design.

The current study had some limitations should be taken into consideration in future research. Relatively small sample size, as noted in certain clinical subgroups (e.g., musculoskeletal involvement, n=6), may affect the ability to detect additional associations. The cross-sectional design prevents assessment of FSTL1’s predictive utility for disease flares or treatment response. Our cohort included patients already receiving systemic immunosuppressants therapy at the time of sampling, which could affect FSTL1 production or clearance, altering serum FSTL1 levels independent of disease activity. Hence, the current findings should be interpreted as reflecting serum levels of FSTL1 in BD treated patients rather than in untreated active one.

## CONCLUSION AND RECOMMENDATIONS

Based on our findings, we recommend the following points for future research, prospective longitudinal studies and to include treatment-naïve patients to evaluate the predictive ability of FSTL1 in disease flares, monitoring treatment response and better explore the primary inflammatory role of FSTL1, and studying FSTL1 with other potential inflammatory markers in order to improve its diagnostic and prognostic accuracy. In conclusion, this study provides promising evidence that FSTL1 serum levels are significantly increased in BD and are associated with both activity and cumulative organ damage, may serve as a biomarker reflecting the chronic inflammation characteristic of BD. While serum FSTL1 level demonstrates limited utility as a standalone diagnostic tool, its high specificity and its correlations with some validated disease measures may suggest its potential clinical value as part of combined biomarker panels for BD monitoring.

## Data Availability

Data is available from the corresponding author upon reasonable request.
